# Supplementary material for: Ancestral perinatal obesogen exposure results in a transgenerational thrifty phenotype in mice
Source: Nat Commun. 2017 Dec 8;8:2012. doi: 10.1038/s41467-017-01944-z (PMC5722856; doi:10.1038/s41467-017-01944-z)
Supplement: Supplementary file 1 — Supplementary Information [file 41467_2017_1944_MOESM1_ESM.pdf]

Supplementary Figure 1

A.

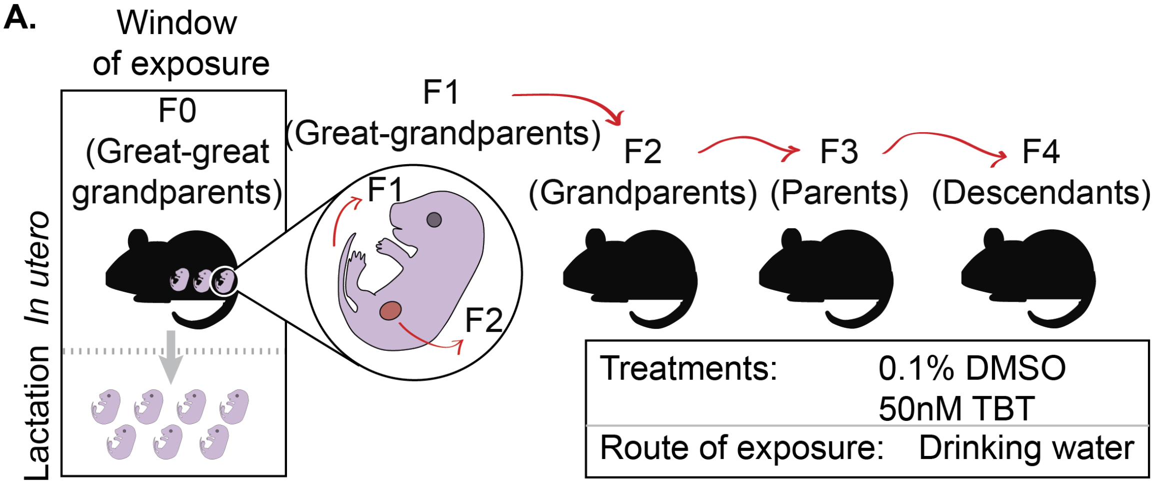

B.

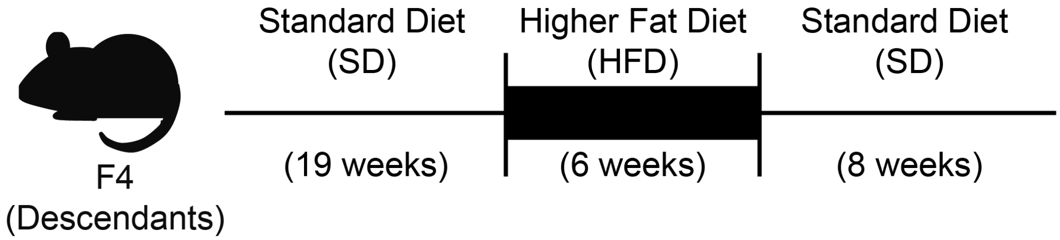

% Kcal	Standard Diet (SD)	Higher Fat Diet (HFD)
Fat	13.2	21.2
Protein	24.6	23.2
Carbohydrates	62.1	55.2
Energy (kcal/g)	3.07	3.56

C.

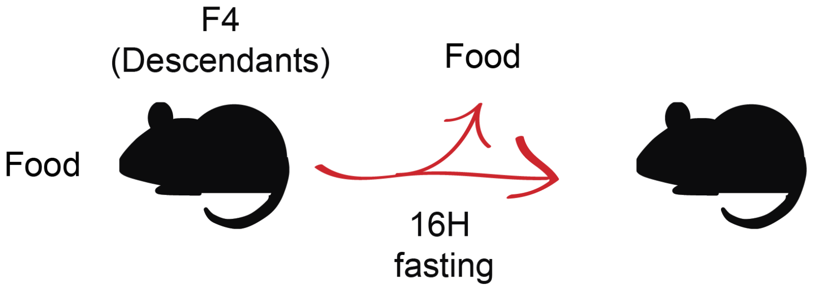

Supplementary Fig. 1. Schematic representation of the experimental design. (**A**) Window of exposure and treatment provided to the mice. (**B**) Diet challenge. (**C**) Fasting challenge.

Supplementary Figure 2

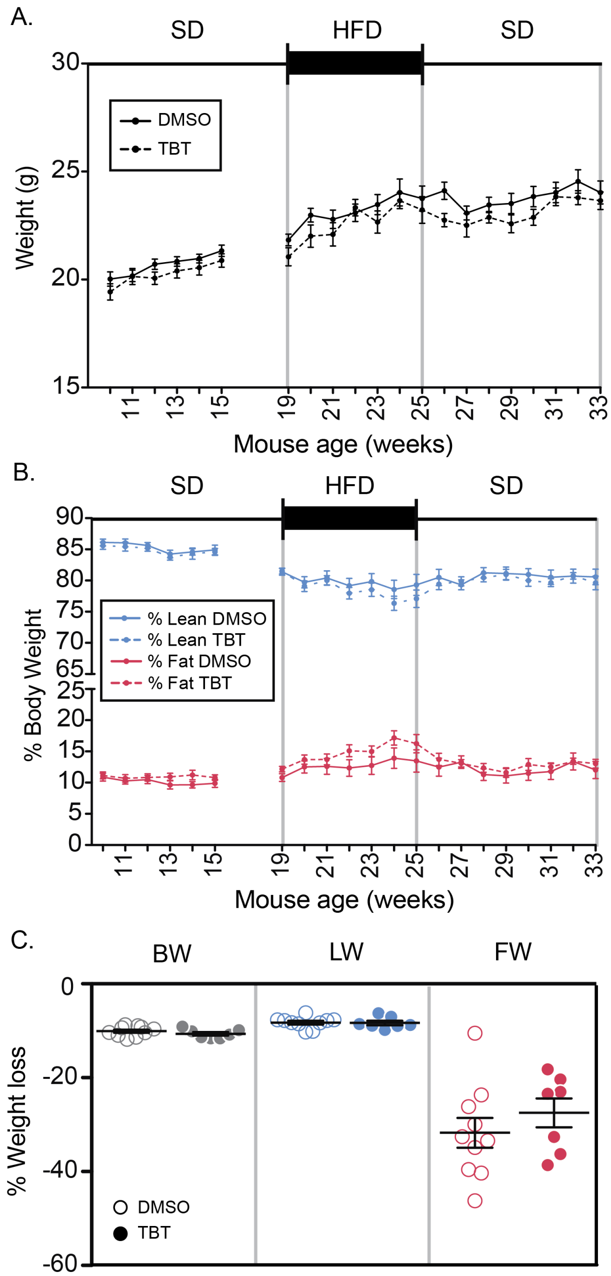

Supplementary Fig. 2. Effect of metabolic challenges in F4 females after ancestral exposure to TBT. Body weight (**A**) and relative body composition (**B**) of DMSO (n=10) and TBT (n=7) females throughout the course of the experiment. (**C**) Percentage of body weight (BW), lean weight (LW) and fat weight (FW) loss after fasting in DMSO (n=10) and TBT (n=7) females. Statistical significance was determined using two-way ANOVA in panels A-B and one-way ANOVA in panel C. Pair-wise Bonferroni post-tests were used to compare different groups in all panels. Data is presented as mean ± s.e.m.

Supplementary Figure 3

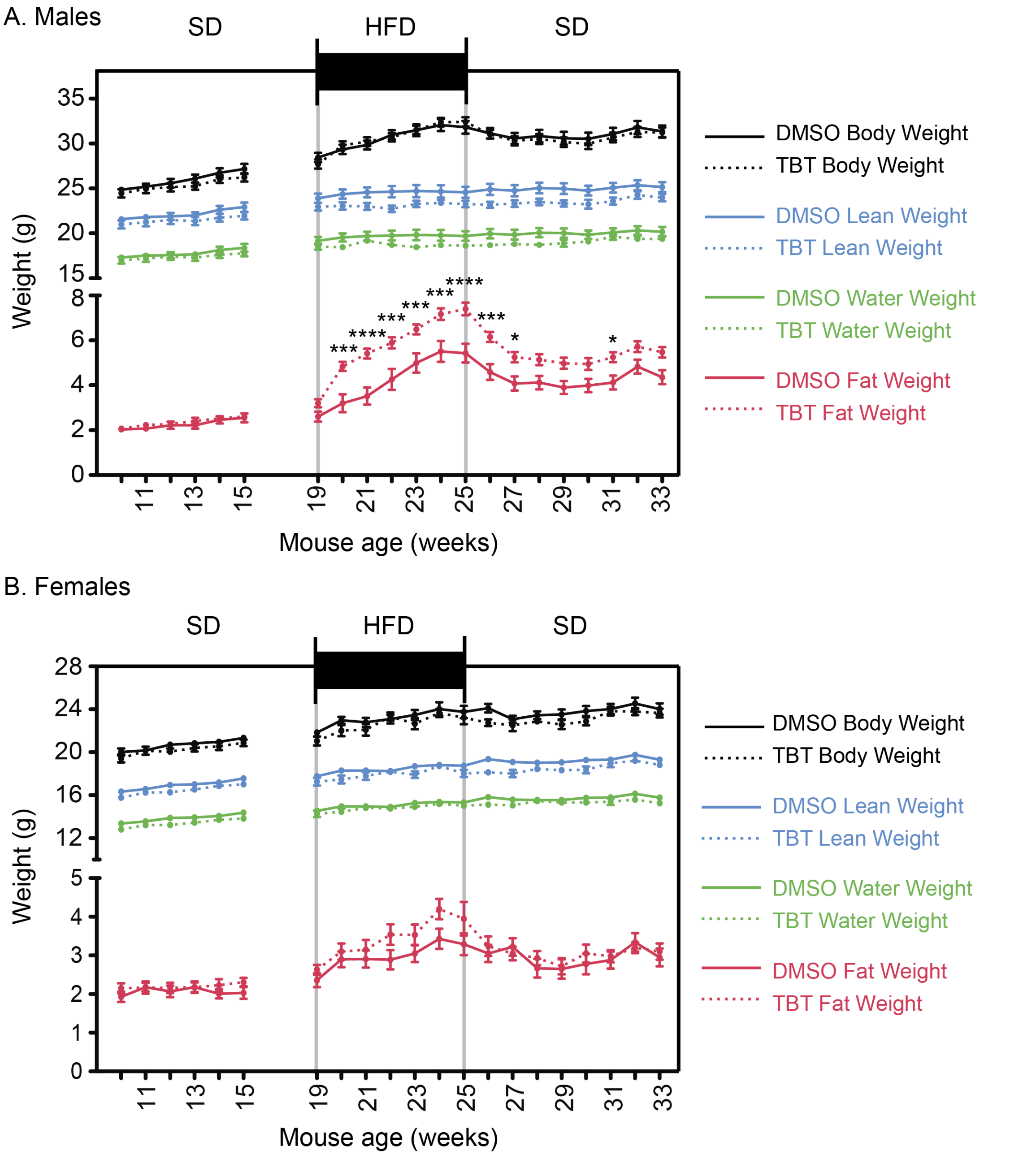

Supplementary Fig. 3. Total body, lean, water and fat weight. Body weight, lean, total water and fat weight were represented for both males (n ≥ 10) (**A**) and females (n ≥ 7) (**B**). Body composition was measured using EchoMRI^{TM} Whole Body Composition Analyzer, which provides lean, fat and water content. Total water weight includes free water mainly from the bladder and water contained in lean. Statistical significance in all panels was determined using two-way ANOVA and pair-wise Bonferroni post-tests to compare different groups. Data is presented as mean ± s.e.m. * $p < 0.05$; ** $p < 0.01$; *** $p < 0.001$; **** $p < 0.0001$.

Supplementary Figure 4

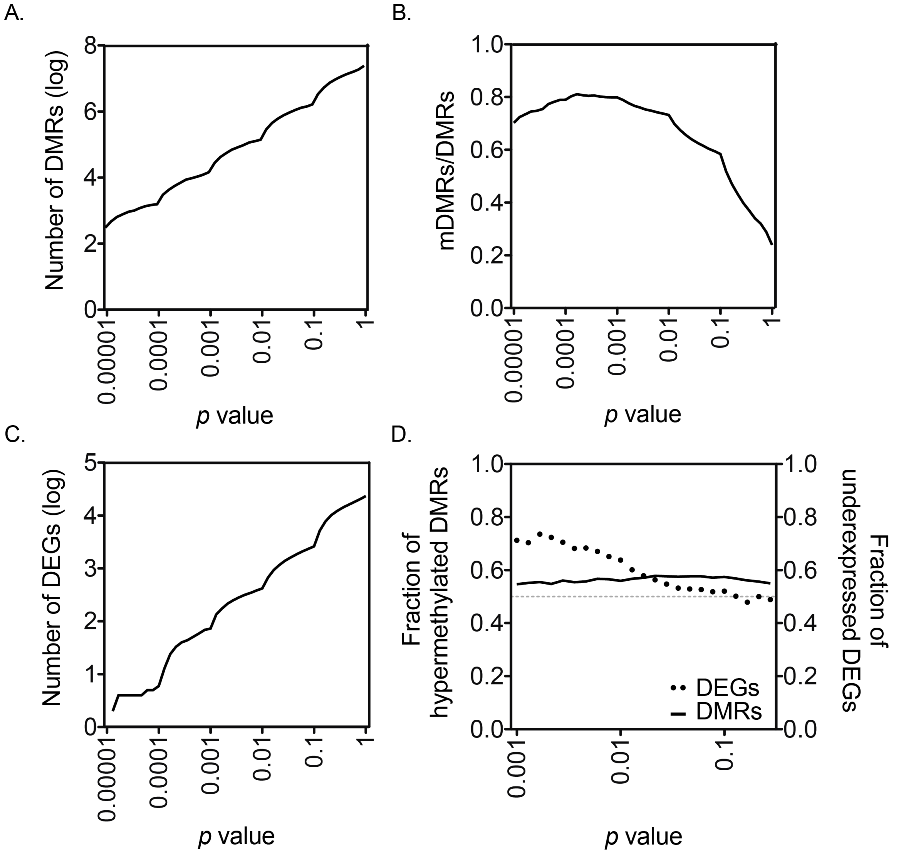

Supplementary Fig. 4. Summary for TBT-dependent DNA methylome and transcriptome variation. (**A**) Number of Differentially Methylated Regions (DMRs) at any given *p* value. DMRs represent whole-genome consecutive, non-overlapping 100 bp windows with significantly different MBD-seq read coverage between TBT and DMSO samples. (**B**) Rate of new discoveries independency. Merged DMRs (mDMRs) result after merging adjacent DMRs with the same direction of change at any given *p* value. The mDMRs/DMRs ratio was used as a measure of new discoveries independency. The mDMRs/DMRs ratio would increase when newly discovered DMRs upon significance relaxation tended to be independent from already identified DMRs. The mDMRs/DMRs ratio would decrease when newly discovered DMRs upon significance relaxation tended to be adjacent to already identified DMRs. (**C**) Number of Differentially Expressed Genes (DEGs) at any given *p* value. DEGs represent genes with significantly different RNA-seq read coverage for TBT and DMSO samples. (**D**) Direction of change bias for TBT-dependent DNA methylome and transcriptome variation. The fraction of hypermethylated DMRs represents the fraction of DMRs with significantly higher MBD-seq read coverage in TBT than in DMSO samples divided by the total number of DMRs. The fraction of underexpressed DEGs represent DEGs with significantly lower RNA-seq read coverage in TBT than in DMSO samples divided by the total number of DEGs.

Supplementary Figure 5

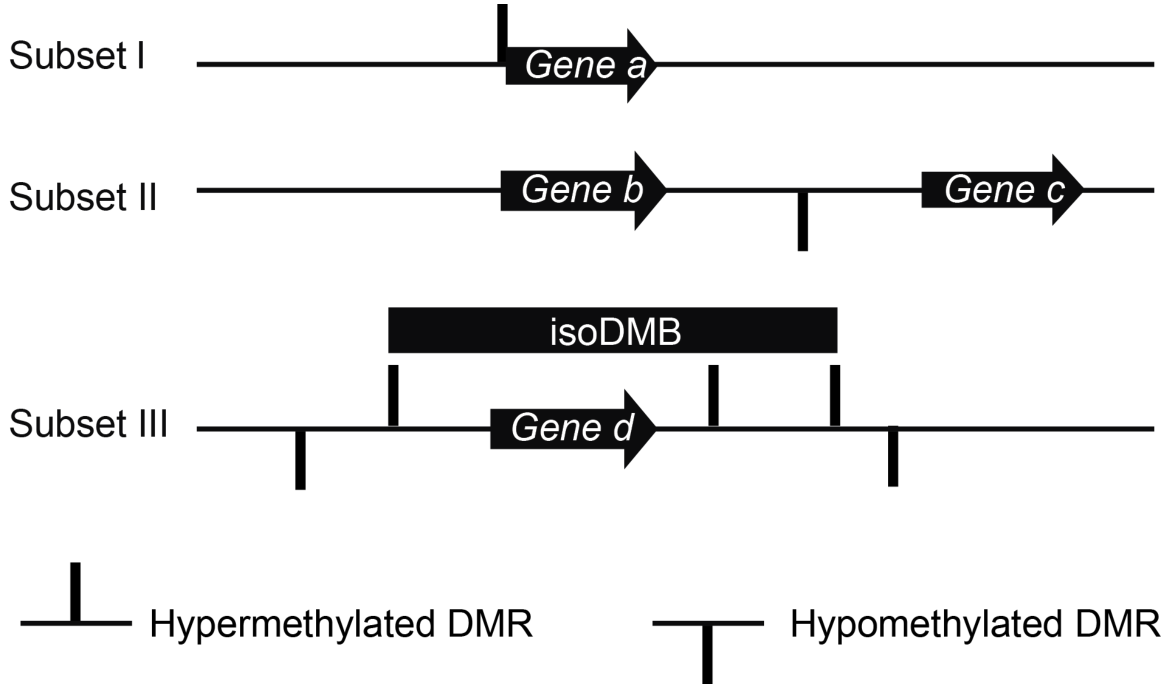

Supplementary Fig. 5. Subsets of mouse genes defined by their association with TBT-dependent DNA methylation changes in F4 male gWAT. TBT-dependent DNA methylome variation structures and their gene associations. Differentially Methylated Regions (DMRs) represent whole-genome consecutive, non-overlapping 100 bp windows with significantly different MBD-seq read coverage between TBT and DMSO samples. iso-Differentially Methylated Blocks (isoDMBs) represent chromosome regions punctuated by iso-directional DMRs. Three subsets of genes were defined depending on their association with DMR or isoDMBs. Subset I encompasses genes with at least one DMR in the close vicinity of their transcription start site (TSS; from -1,500 bp to 500 bp from the TSS). Subset II encompasses genes overlapping or flanking at least one DMR regardless of its distance to the gene TSS. Subset III encompasses genes located within isoDMBs.

Supplementary Figure 6

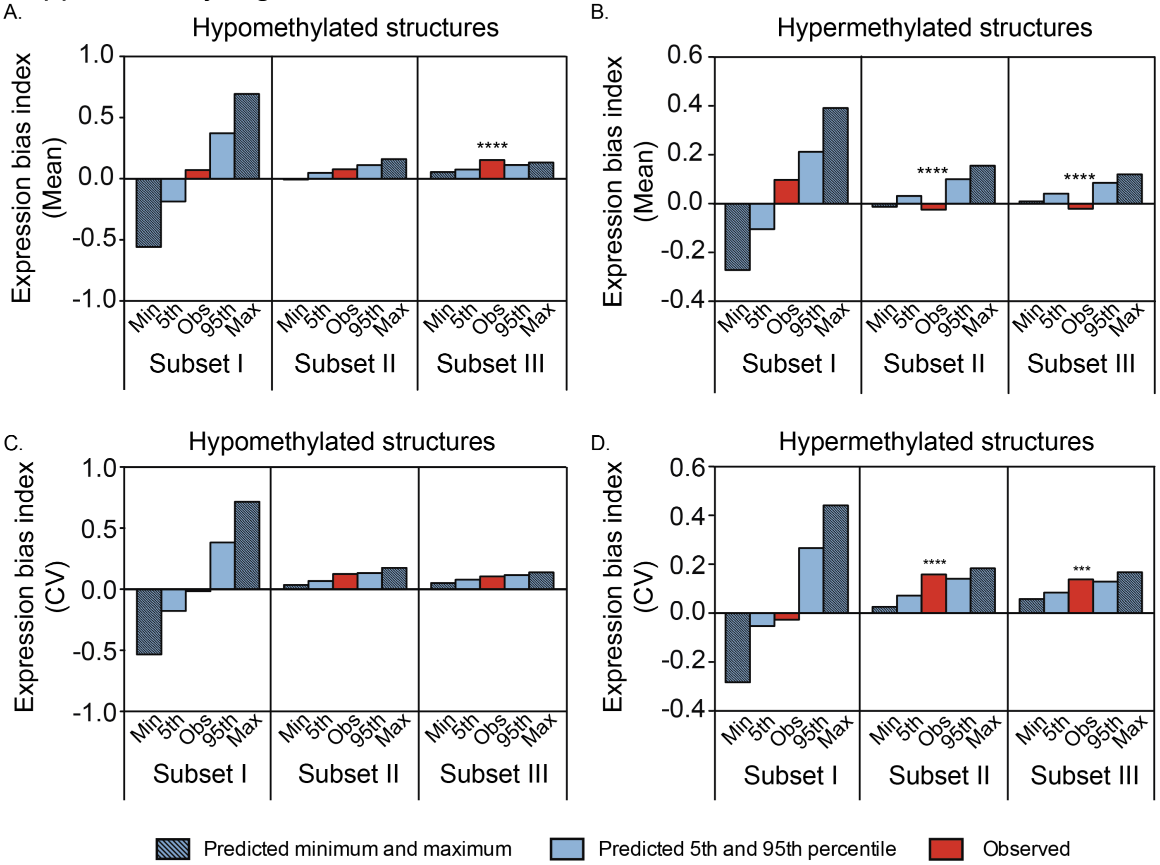

Supplementary Fig. 6. Study of the coordination of TBT-dependent DNA methylome and transcriptome changes using Monte Carlo-Wilcoxon matched-pairs signed-ranks tests (MCW tests). For each subset of genes defined by their association to DMRs or isoDMBs (Supplementary Fig. 5), and DMR direction of change, we performed MCW tests using F4 male gWAT RNA-seq data from TBT and DMSO samples. We performed MCW tests independently for transcript abundance mean (**A-B**) and coefficient of variation (CV) (**C-D**). Gene expression bias index quantifies general biases in gene expression measures for the subset of genes under study considering transcript abundance information for all genes interrogated using RNA-seq (see Methods). Positive gene expression bias indexes indicate that gene expression measures tend to be higher in TBT than in DMSO samples, whereas negative gene expression bias indexes indicate that gene expression measures tend to be lower in TBT than in DMSO samples. Gene expression bias indexes were calculated before and after randomly rearranging subset tags 10,000 times respecting chromosome assortment. The distribution of predicted gene expression bias indexes using random permutations was represented using their minimum, 5th and 95th percentiles, and maximum. *p* values represent the fraction of random permutations were predicted gene expression bias indexes resulted more extreme than observed ones. ***<0.001, **** *p*<0.0001.

Supplementary Figure 7

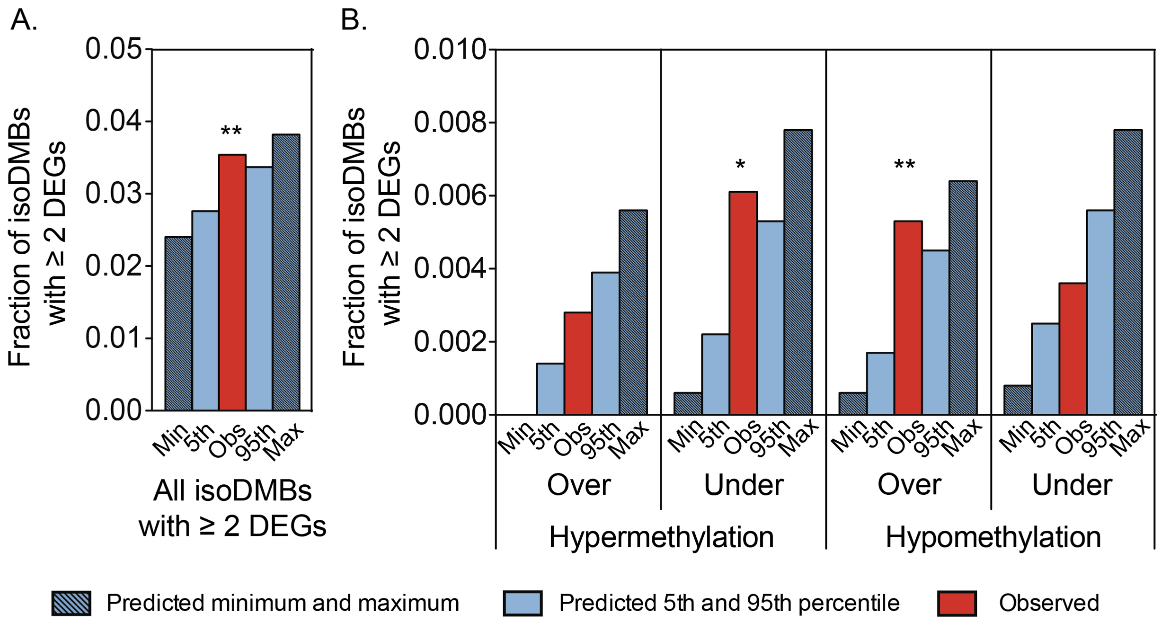

Supplementary Fig. 7. Distribution of groups of TBT-dependent Differentially Expressed Genes (DEGs) with regard to TBT-dependent iso-Differentially Methylated Blocks (isoDMBs). The fraction of isoDMBs containing at least two DEGs (isoDMBs with ≥2 DEGs) was calculated before and after randomly rearranging DEG tags 10,000 times respecting their chromosome assortment. The distribution of predicted fractions of isoDMBs with ≥2 DEGs using random permutations were represented using their minimum, 5th and 95th percentiles, and maximum. Chart **A** represents the fraction of isoDMBs with ≥2 DEGs regardless of their direction of change, and chart **B** results or each potential combination of DEG and isoDMB direction of change. *p* values represent the fraction of random permutations were predicted fractions of isoDMBs with ≥2 DEGs resulted more extreme than observed ones. **p*<005, ** *p*<0.01.

Supplementary Figure 8

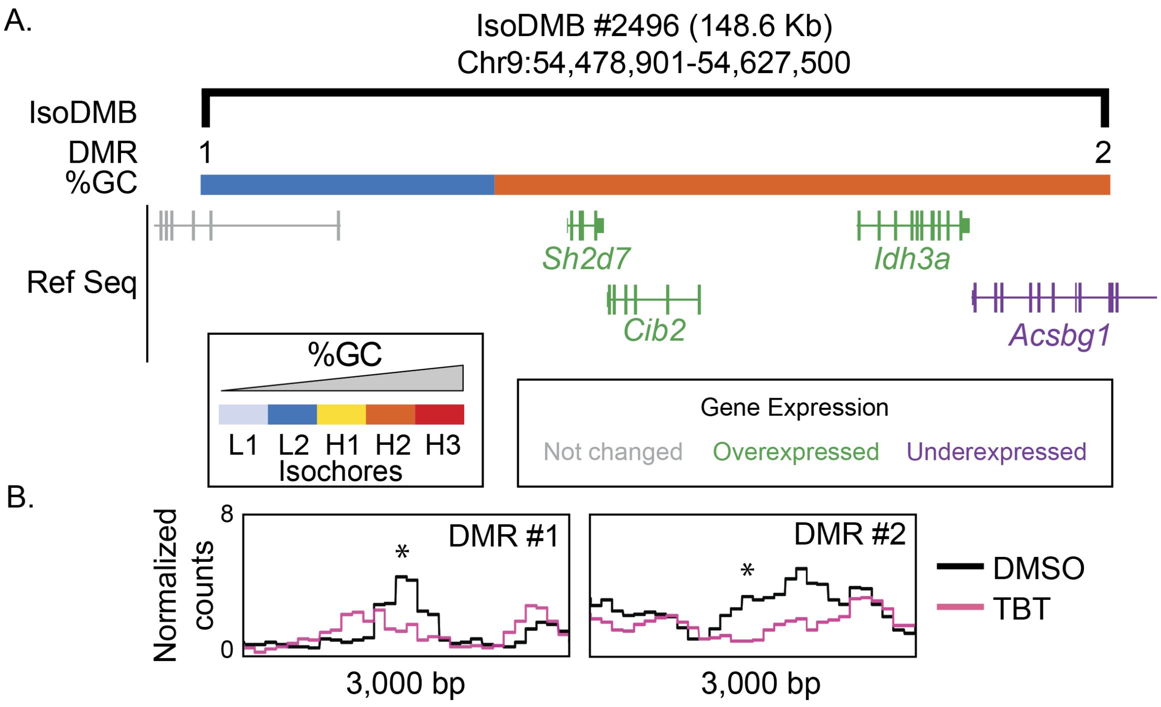

Supplementary Fig. 8. Schematic representation of isoDMB #2496. (**A**) Upper panel represents isoDMB #2496 (black bar) and the GC content of the overlapping region. Hypomethylated differentially methylated regions (DMRs) are represented with black vertical bars and numbered (1-2). Genes located within the isoDMB region colored in green and purple are found overexpressed and underexpressed in our data set, respectively. Grey genes represent genes within the isoDMB whose expression does not change between TBT and DMSO samples. (**B**) Bottom panels show the variation for the mean (n=4) of MBD-seq reads coverage for TBT and DMSO samples within 3,000 bp regions with the 100 bp DMR indicated with an asterisk.

Supplementary Figure 9

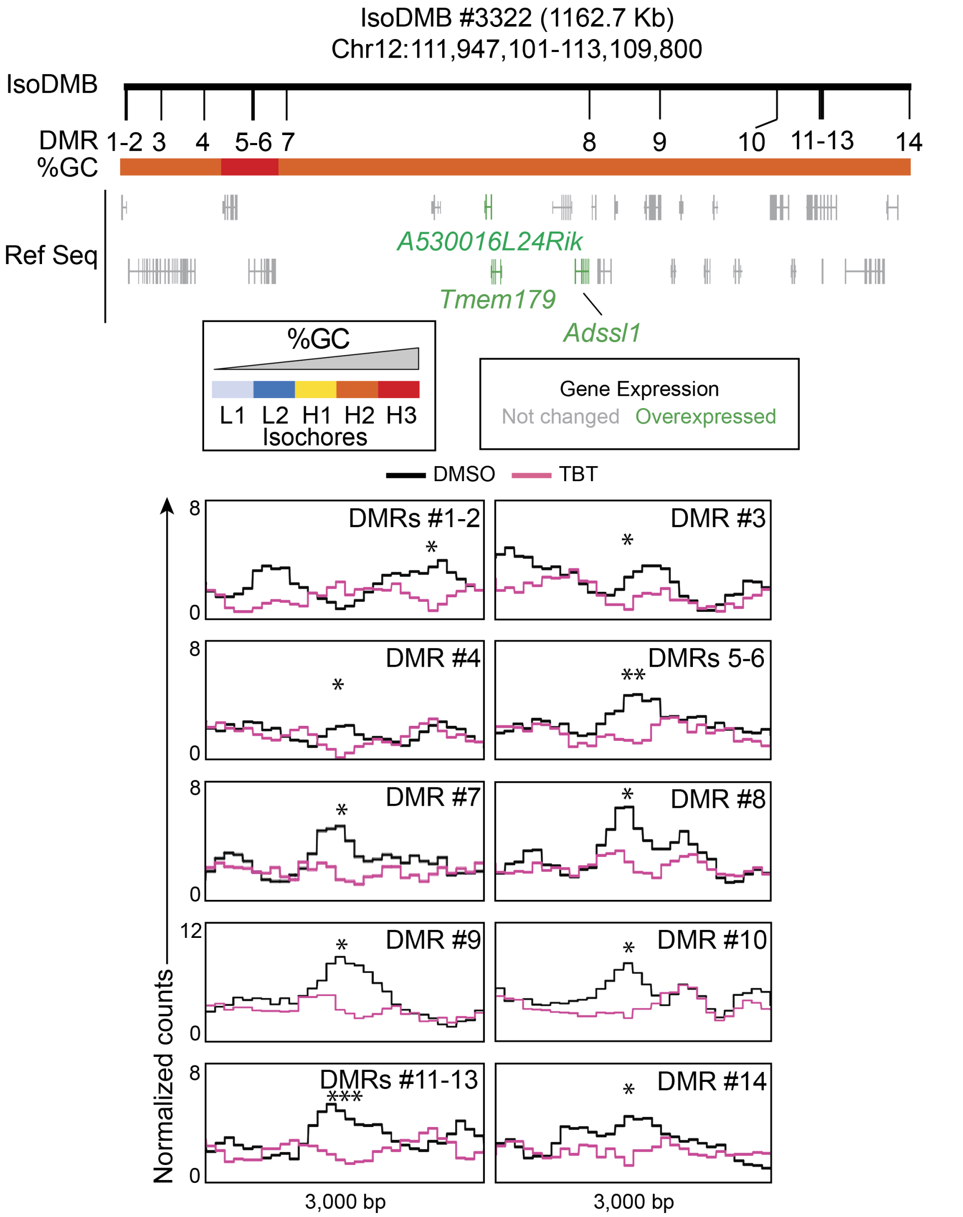

Supplementary Fig. 9. Schematic representation of isoDMB #3322. (**A**) Upper panel represents isoDMB #3322 (black bar) and the GC content of the overlapping region. Hypomethylated differentially methylated regions (DMRs) are represented with black vertical bars and numbered (1-14). Genes located within the region spanned by the isoDMB colored in green are found overexpressed in our data set. Grey genes represent genes within the isoDMB whose expression does not change between TBT and DMSO samples. (**B**) Bottom panels show the variation for the mean (n=4) of MBD-seq reads coverage for TBT and DMSO samples within 3,000 bp regions with the 100 bp DMR indicated with an asterisk.

Supplementary Figure 10

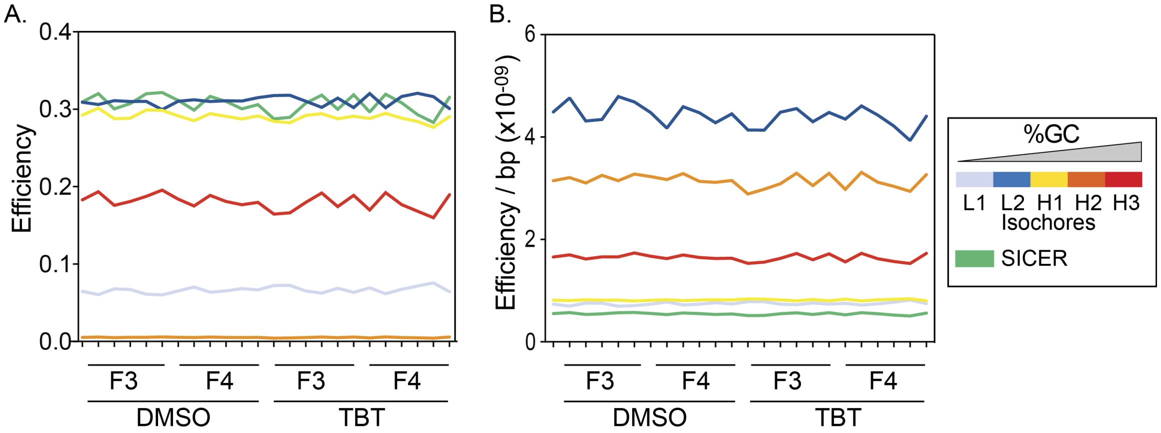

Supplementary Fig. 10. Sample efficiency of ATAC-seq analyses for DMSO and TBT sperm samples. ATAC-seq was used to compare chromatin accessibility patterns for 24 single-male samples of sperm (2 generations X 2 treatments X 6 biological replicates). The similarity between samples was inspected using chromVar guided by SICER islands and DNA base composition. (A) For each sample, sample efficiency was calculated as the number of ATAC-seq reads mapping within SICER islands or the five regions defined by based composition divided by the total number of reads for each sample. (B) Normalized sample efficiencies using the cumulative length of the five regions with different base composition (efficiency / bp).

Supplementary Figure 11

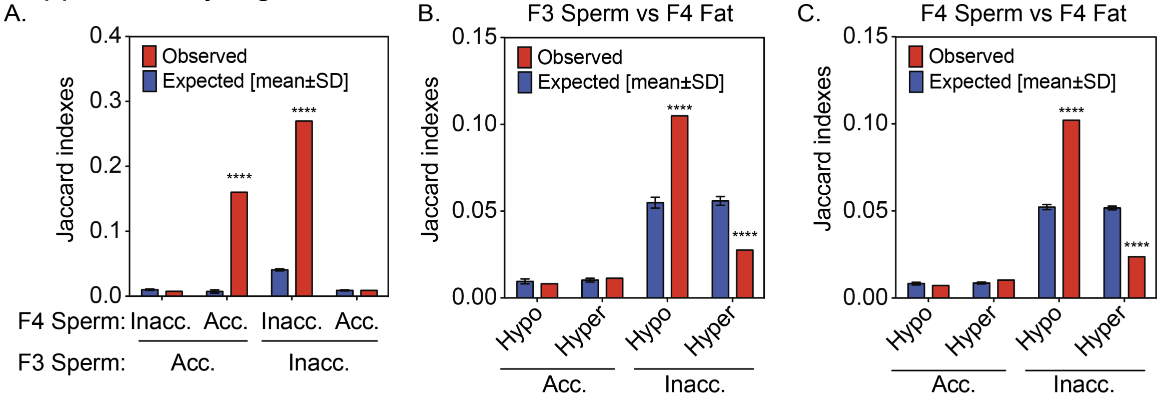

Supplementary Fig 11. Overlap for SICER islands showing differential chromatin accessibility between sperm of F3 and F4 generations, and each one of them with F4 adipose isoDMBs. SICER was used to define chromosome regions with significant differential chromatin accessibility using ATAC-seq reads (n=6 per treatment group and generation) (see Methods for further details). SICER islands for which ATAC-seq read coverage was larger in TBT than in DMSO samples were deemed as accessible (Acc.), whereas SICER islands for which ATAC-seq read coverage was larger in DMSO than in TBT samples were deemed as inaccessible (Inacc.). Hyper Browser was used to calculate the overlap between F3 and F4 SICER islands, and each of them with F4 adipose isoDMBs, and to test their statistical significance using Monte Carlo tests[64]. The Jaccard index that measures the similarity between datasets was used to quantify the general overlap between F3 and F4 DAIs and with F4 adipose isoDMBs (see Methods). Expected Jaccard indexes were calculated after randomly rearranging isoDMBs location 10,000 times respecting isoDMBs, and DAIs length and chromosome assortment. *p* values represent the number of random simulations showing Jaccard indexes more extreme than observed ones. Expected data is presented as mean ± s.d.** p<0.01, *** p<0.001, **** p<0.0001.

Supplementary Table 1. Total weight and fraction of fat depots in F1-F4 females

	FEMALES				
	DMSO (n=15)		50nM TBT (n=15)		
FIRST GENERATION	Mean	SEM	Mean	SEM	*p* value[1]
Body weight (g)	17.3	0.219	18	0.224	0.042
Gonadal fat (%)	0.87	0.051	0.94	0.068	0.442
Peri-renal (%)	0.32	0.025	0.38	0.03	0.045
Interscapular white (%)	0.34	0.028	0.34	0.01	0.989
Inguinal (%)	0.95	0.046	1.04	0.046	0.342
Interscapular brown (%)	0.28	0.007	0.29	0.015	0.558
SECOND GENERATION					
Body weight (g)	17.5	0.176	17.8	0.296	0.37
Gonadal fat (%)	0.9	0.033	0.93	0.053	0.636
Peri-renal (%)	0.37	0.024	0.36	0.02	0.945
Interscapular white (%)	0.32	0.018	0.29	0.022	0.313
Inguinal (%)	1.22	0.046	1.28	0.047	0.333
Interscapular brown (%)	0.29	0.006	0.29	0.009	0.976
THIRD GENERATION					
Body weight (g)	17.6	0.171	17.5	0.176	0.627
Gonadal fat (%)	1.04	0.05	0.96	0.054	0.282
Peri-renal (%)	0.39	0.021	0.37	0.021	0.536
Interscapular white (%)	0.25	0.018	0.26	0.018	0.497
Inguinal (%)	1.29	0.05	1.3	0.043	0.888
Interscapular brown (%)	0.27	0.006	0.31	0.01	0.009
FOURTH GENERATION					
Body weight (g)	17	0.221	17.3	0.118	0.404
Gonadal fat (%)	1.12	0.082	1.06	0.046	0.541
Peri-renal (%)	0.43	0.035	0.43	0.022	0.938
Interscapular white (%)	0.35	0.03	0.31	0.021	0.401
Inguinal (%)	1.43	0.091	1.31	0.063	0.294
Interscapular brown (%)	0.29	0.008	0.28	0.008	0.551

[1]Unpaired *t-test*

Supplementary Table 2. Total weight and fraction of fat depots in F1-F4 males

	MALES				
	DMSO (n=15)		50nM TBT (n=15)		
FIRST GENERATION	Mean	SEM	Mean	SEM	*p* value[1]
Body weight (g)	20.6	0.212	21.4	0.292	0.033
Gonadal fat (%)	1.09	0.033	1.08	0.033	0.879
Peri-renal (%)	0.26	0.015	0.26	0.022	0.978
Interscapular white (%)	0.27	0.023	0.27	0.026	0.994
Inguinal (%)	0.91	0.041	0.89	0.036	0.671
Interscapular brown (%)	0.26	0.008	0.27	0.01	0.47
SECOND GENERATION					
Body weight (g)	22.1	0.298	22.2	0.302	0.922
Gonadal fat (%)	0.91	0.045	1.1	0.044	0.005
Peri-renal (%)	0.22	0.019	0.27	0.014	0.044
Interscapular white (%)	0.17	0.013	0.22	0.013	0.034
Inguinal (%)	0.81	0.041	0.92	0.031	0.052
Interscapular brown (%)	0.24	0.011	0.25	0.008	0.246
THIRD GENERATION					
Body weight (g)	21.9	0.206	21.9	0.311	0.952
Gonadal fat (%)	1	0.03	1.14	0.031	0.003
Peri-renal (%)	0.24	0.01	0.31	0.013	0.0003
Interscapular white (%)	0.19	0.012	0.25	0.014	0.001
Inguinal (%)	0.95	0.037	1.09	0.027	0.003
Interscapular brown (%)	0.25	0.006	0.28	0.005	0.0005
FOURTH GENERATION					
Body weight (g)	21.5	0.294	22.4	0.399	0.079
Gonadal fat (%)	1.07	0.029	1.15	0.022	0.038
Peri-renal (%)	0.23	0.016	0.28	0.021	0.119
Interscapular white (%)	0.23	0.021	0.19	0.008	0.066
Inguinal (%)	1	0.029	0.88	0.039	0.023
Interscapular brown (%)	0.24	0.012	0.22	0.01	0.206

[1]Unpaired *t-test*

Supplementary Table 3. Leptin plasma levels in F4 males and females descendants of DMSO- and TBT-treated animals at 33 weeks of age.

	DMSO-lineage					TBT-lineage					
**Gender**	**N**	**Mean**	**SD**	**SEM**	**CV**	**N**	**Mean**	**SD**	**SEM**	**CV**	**Unpaired t-test *p* value**
Male	10	2039.7	967.70	306.0147262	47.44	12	3522.50	1289.9	372.35	36.62	0.0071
Female	10	1138.1	1178.17	372.5712467	103.52	7	2340.39	1494.2	564.74	63.84	0.0830

Supplementary Table 4. Protein plasma levels in F4 descendants of DMSO- and TBT-treated animals at 33 weeks of age.

	DMSO-lineage					TBT-lineage					
**Protein concentration**	**N**	**Mean**	**SD**	**SEM**	**CV**	**N**	**Mean**	**SD**	**SEM**	**CV**	**Unpaired t-test *p* value**
Adiponectin (ug/ml)	8	12.3	0.51	0.16	4.15	8	12.50	0.7	0.19	5.38	0.5100
Ghrelin (pg/ml)	8	1359.0	792.60	280.2	58.32	8	2030.00	1161.0	410.50	57.20	0.1980
GIP (pg/ml)	8	104.5	18.53	6.55	17.73	8	110.90	17.4	6.16	15.71	0.4922
GLP-1 (pg/ml)	8	14.8	3.50	1.32	23.66	8	18.87	6.7	2.36	35.40	0.1702
Glucagon (pg/ml)	8	40.2	7.74	2.92	19.28	8	47.45	10.9	3.84	22.87	0.1634
Insulin (pg/ml)	8	487.6	146.60	51.84	30.07	8	507.20	216.5	76.54	42.68	0.8352
PAI-1 (pg/ml)	8	251.3	86.28	30.5	34.34	8	267.30	61.1	21.59	22.85	0.6748
Resistin (pg/ml)	8	24.6	5.57	1.97	22.66	8	24.71	6.9	2.43	27.86	0.9668

Supplementary Table 5. Number of F1-F4 parents and descendants

	Females bred		Pregnant females		Pups born		Pups weaned		Females weaned		Males weaned	
Generation	DMSO	TBT	DMSO	TBT	DMSO	TBT	DMSO	TBT	DMSO	TBT	DMSO	TBT
F1	25	25	20	19	130	138	98	124	48	67	50	57
F2	30	30	23	26	153	158	110	104	54	48	57	56
F3	25	28	20	26	135	119	113	80	64	32	49	48
F4	30	11	24	10	166	70	124	57	61	26	63	31

Supplementary Methods

Annotated r code for the analysis of TBT-dependent changes on F4 adipose DNA methylome and transcriptome and F3 and F4 sperm chromatin accessibility.

1. Use of MEDIPS to identify significant TBT-dependent changes in F4 male adipose tissue DNA methylome

```
  # Requirements: R (3.3), Bioconductor (3.3), MEDIPS (1.22), and
BSgenome.Mmusculus.UCSC.mm10
  # Datasets (Gene Expression Omnibus: GSE105051):
Met_DMSO_1.merged.sorted.uniq.bam (GSM2816966),
Met_DMSO_2.merged.sorted.uniq.bam (GSM2816967),
Met_DMSO_3.merged.sorted.uniq.bam (GSM2816968),
Met_DMSO_4.merged.sorted.uniq.bam (GSM2816969),
Met_TBT_1.merged.sorted.uniq.bam (GSM2816970),
Met_TBT_2.merged.sorted.uniq.bam (GSM2816971),
Met_TBT_3.merged.sorted.uniq.bam (GSM2816972),
Met_TBT_4.merged.sorted.uniq.bam (GSM2816973)
  # Setting MEDIPS parameters

    BSgenome = "BSgenome.Mmusculus.UCSC.mm10"
    uniq = 1e-3
    extend = 300
    shift = 0
    ws = 100
    selectedchromosomes = c("chr1", "chr2", "chr3", "chr4", "chr5",
"chr6", "chr7", "chr8", "chr9", "chr10", "chr11", "chr12", "chr13",
"chr14", "chr15", "chr16", "chr17", "chr18", "chr19", "chrX", "chrY",
"chrM")

  # Creating MEDIPS sets from bam files

    fat.DMSO1.MeDIP <- MEDIPS.createSet(file =
"Meth_DMSO_1.merged.sorted.uniq.bam", BSgenome = BSgenome, extend =
extend, shift = shift, uniq = uniq, window_size = ws, chr.select =
selectedchromosomes)
    fat.DMSO2.MeDIP <- MEDIPS.createSet(file =
"Meth_DMSO_2.merged.sorted.uniq.bam", BSgenome = BSgenome, extend =
extend, shift = shift, uniq = uniq, window_size = ws, chr.select =
selectedchromosomes)
    fat.DMSO3.MeDIP <- MEDIPS.createSet(file =
"Meth_DMSO_3.merged.sorted.uniq.bam", BSgenome = BSgenome, extend =
extend, shift = shift, uniq = uniq, window_size = ws, chr.select =
selectedchromosomes)
    fat.DMSO4.MeDIP <- MEDIPS.createSet(file =
"Meth_DMSO_4.merged.sorted.uniq.bam", BSgenome = BSgenome, extend =
extend, shift = shift, uniq = uniq, window_size = ws, chr.select =
selectedchromosomes)
    fat.DMSO.MeDIP <- c(fat.DMSO1.MeDIP, fat.DMSO2.MeDIP,
fat.DMSO3.MeDIP, fat.DMSO4.MeDIP)
```

```
    fat.TBT1.MeDIP <- MEDIPS.createSet(file =
"Meth_TBT_1.merged.sorted.uniq.bam", BSgenome = BSgenome, extend =
extend, shift = shift, uniq = uniq, window_size = ws, chr.select =
selectedchromosomes)
    fat.TBT2.MeDIP <- MEDIPS.createSet(file =
"Meth_TBT_2.merged.sorted.uniq.bam", BSgenome = BSgenome, extend =
extend, shift = shift, uniq = uniq, window_size = ws, chr.select =
selectedchromosomes)
    fat.TBT3.MeDIP <- MEDIPS.createSet(file =
"Meth_TBT_3.merged.sorted.uniq.bam", BSgenome = BSgenome, extend =
extend, shift = shift, uniq = uniq, window_size = ws, chr.select =
selectedchromosomes)
    fat.TBT4.MeDIP <- MEDIPS.createSet(file =
"Meth_TBT_4.merged.sorted.uniq.bam", BSgenome = BSgenome, extend =
extend, shift = shift, uniq = uniq, window_size = ws, chr.select =
selectedchromosomes)
    fat.TBT.MeDIP <- c(fat.TBT1.MeDIP, fat.TBT2.MeDIP, fat.TBT3.MeDIP,
fat.TBT4.MeDIP)

  # Creating a coupling vector
    CS <- MEDIPS.couplingVector(pattern = "CG", refObj =
fat.DMSO1.MeDIP)

  # Comparing MDB-seq read coverage for Mmusculus mm10 100 bp-genomic
windows
    dmr.fat.TBT <- MEDIPS.meth(MSet1 = fat.TBT.MeDIP, MSet2 =
fat.DMSO.MeDIP, CSet = CS, p.adj = "fdr", diff.method = "edgeR", MeDIP
= FALSE, CNV = FALSE, minRowSum = 10, diffnorm = "quantile")

  # Setting a significance threshold using significant DMRs and merged
DMRs at different p values
    data <- dmr.fat.TBT
    p.value <-
c("0.00001","0.00002","0.00003","0.00004","0.00005","0.00006","0.00007
","0.00008","0.00009","0.0001","0.0002","0.0003","0.0004","0.0005","0.
0006","0.0007","0.0008","0.0009","0.001","0.002","0.003","0.004","0.00
5","0.006","0.007","0.008","0.009","0.01","0.02","0.03","0.04","0.05",
"0.06","0.07","0.08","0.09","0.1","0.2","0.3","0.4","0.5","0.6","0.7",
"0.8","0.9","1")
    summary <- data.frame(p.value)

    summary$dmr.fat.hyper <- c(
      sum(data$edgeR.logFC>0 & data$edgeR.p.value<0.00001,na.rm=TRUE),
      sum(data$edgeR.logFC>0 & data$edgeR.p.value<0.00002,na.rm=TRUE),
      sum(data$edgeR.logFC>0 & data$edgeR.p.value<0.00003,na.rm=TRUE),
      sum(data$edgeR.logFC>0 & data$edgeR.p.value<0.00004,na.rm=TRUE),
      sum(data$edgeR.logFC>0 & data$edgeR.p.value<0.00005,na.rm=TRUE),
      sum(data$edgeR.logFC>0 & data$edgeR.p.value<0.00006,na.rm=TRUE),
      sum(data$edgeR.logFC>0 & data$edgeR.p.value<0.00007,na.rm=TRUE),
```

```
  sum(data$edgeR.logFC>0 & data$edgeR.p.value<0.00008,na.rm=TRUE),
  sum(data$edgeR.logFC>0 & data$edgeR.p.value<0.00009,na.rm=TRUE),
  sum(data$edgeR.logFC>0 & data$edgeR.p.value<0.0001,na.rm=TRUE),
  sum(data$edgeR.logFC>0 & data$edgeR.p.value<0.0002,na.rm=TRUE),
  sum(data$edgeR.logFC>0 & data$edgeR.p.value<0.0003,na.rm=TRUE),
  sum(data$edgeR.logFC>0 & data$edgeR.p.value<0.0004,na.rm=TRUE),
  sum(data$edgeR.logFC>0 & data$edgeR.p.value<0.0005,na.rm=TRUE),
  sum(data$edgeR.logFC>0 & data$edgeR.p.value<0.0006,na.rm=TRUE),
  sum(data$edgeR.logFC>0 & data$edgeR.p.value<0.0007,na.rm=TRUE),
  sum(data$edgeR.logFC>0 & data$edgeR.p.value<0.0008,na.rm=TRUE),
  sum(data$edgeR.logFC>0 & data$edgeR.p.value<0.0009,na.rm=TRUE),
  sum(data$edgeR.logFC>0 & data$edgeR.p.value<0.001,na.rm=TRUE),
  sum(data$edgeR.logFC>0 & data$edgeR.p.value<0.002,na.rm=TRUE),
  sum(data$edgeR.logFC>0 & data$edgeR.p.value<0.003,na.rm=TRUE),
  sum(data$edgeR.logFC>0 & data$edgeR.p.value<0.004,na.rm=TRUE),
  sum(data$edgeR.logFC>0 & data$edgeR.p.value<0.005,na.rm=TRUE),
  sum(data$edgeR.logFC>0 & data$edgeR.p.value<0.006,na.rm=TRUE),
  sum(data$edgeR.logFC>0 & data$edgeR.p.value<0.007,na.rm=TRUE),
  sum(data$edgeR.logFC>0 & data$edgeR.p.value<0.008,na.rm=TRUE),
  sum(data$edgeR.logFC>0 & data$edgeR.p.value<0.009,na.rm=TRUE),
  sum(data$edgeR.logFC>0 & data$edgeR.p.value<0.01,na.rm=TRUE),
  sum(data$edgeR.logFC>0 & data$edgeR.p.value<0.02,na.rm=TRUE),
  sum(data$edgeR.logFC>0 & data$edgeR.p.value<0.03,na.rm=TRUE),
  sum(data$edgeR.logFC>0 & data$edgeR.p.value<0.04,na.rm=TRUE),
  sum(data$edgeR.logFC>0 & data$edgeR.p.value<0.05,na.rm=TRUE),
  sum(data$edgeR.logFC>0 & data$edgeR.p.value<0.06,na.rm=TRUE),
  sum(data$edgeR.logFC>0 & data$edgeR.p.value<0.07,na.rm=TRUE),
  sum(data$edgeR.logFC>0 & data$edgeR.p.value<0.08,na.rm=TRUE),
  sum(data$edgeR.logFC>0 & data$edgeR.p.value<0.09,na.rm=TRUE),
  sum(data$edgeR.logFC>0 & data$edgeR.p.value<0.1,na.rm=TRUE),
  sum(data$edgeR.logFC>0 & data$edgeR.p.value<0.2,na.rm=TRUE),
  sum(data$edgeR.logFC>0 & data$edgeR.p.value<0.3,na.rm=TRUE),
  sum(data$edgeR.logFC>0 & data$edgeR.p.value<0.4,na.rm=TRUE),
  sum(data$edgeR.logFC>0 & data$edgeR.p.value<0.5,na.rm=TRUE),
  sum(data$edgeR.logFC>0 & data$edgeR.p.value<0.6,na.rm=TRUE),
  sum(data$edgeR.logFC>0 & data$edgeR.p.value<0.7,na.rm=TRUE),
  sum(data$edgeR.logFC>0 & data$edgeR.p.value<0.8,na.rm=TRUE),
  sum(data$edgeR.logFC>0 & data$edgeR.p.value<0.9,na.rm=TRUE),
  sum(data$edgeR.logFC>0 & data$edgeR.p.value<2,na.rm=TRUE))

summary$dmr.fat.hypo <- c(
  sum(data$edgeR.logFC<0 & data$edgeR.p.value<0.00001,na.rm=TRUE),
  sum(data$edgeR.logFC<0 & data$edgeR.p.value<0.00002,na.rm=TRUE),
  sum(data$edgeR.logFC<0 & data$edgeR.p.value<0.00003,na.rm=TRUE),
  sum(data$edgeR.logFC<0 & data$edgeR.p.value<0.00004,na.rm=TRUE),
  sum(data$edgeR.logFC<0 & data$edgeR.p.value<0.00005,na.rm=TRUE),
  sum(data$edgeR.logFC<0 & data$edgeR.p.value<0.00006,na.rm=TRUE),
  sum(data$edgeR.logFC<0 & data$edgeR.p.value<0.00007,na.rm=TRUE),
  sum(data$edgeR.logFC<0 & data$edgeR.p.value<0.00008,na.rm=TRUE),
  sum(data$edgeR.logFC<0 & data$edgeR.p.value<0.00009,na.rm=TRUE),
```

```
        sum(data$edgeR.logFC<0 & data$edgeR.p.value<0.0001,na.rm=TRUE),
        sum(data$edgeR.logFC<0 & data$edgeR.p.value<0.0002,na.rm=TRUE),
        sum(data$edgeR.logFC<0 & data$edgeR.p.value<0.0003,na.rm=TRUE),
        sum(data$edgeR.logFC<0 & data$edgeR.p.value<0.0004,na.rm=TRUE),
        sum(data$edgeR.logFC<0 & data$edgeR.p.value<0.0005,na.rm=TRUE),
        sum(data$edgeR.logFC<0 & data$edgeR.p.value<0.0006,na.rm=TRUE),
        sum(data$edgeR.logFC<0 & data$edgeR.p.value<0.0007,na.rm=TRUE),
        sum(data$edgeR.logFC<0 & data$edgeR.p.value<0.0008,na.rm=TRUE),
        sum(data$edgeR.logFC<0 & data$edgeR.p.value<0.0009,na.rm=TRUE),
        sum(data$edgeR.logFC<0 & data$edgeR.p.value<0.001,na.rm=TRUE),
        sum(data$edgeR.logFC<0 & data$edgeR.p.value<0.002,na.rm=TRUE),
        sum(data$edgeR.logFC<0 & data$edgeR.p.value<0.003,na.rm=TRUE),
        sum(data$edgeR.logFC<0 & data$edgeR.p.value<0.004,na.rm=TRUE),
        sum(data$edgeR.logFC<0 & data$edgeR.p.value<0.005,na.rm=TRUE),
        sum(data$edgeR.logFC<0 & data$edgeR.p.value<0.006,na.rm=TRUE),
        sum(data$edgeR.logFC<0 & data$edgeR.p.value<0.007,na.rm=TRUE),
        sum(data$edgeR.logFC<0 & data$edgeR.p.value<0.008,na.rm=TRUE),
        sum(data$edgeR.logFC<0 & data$edgeR.p.value<0.009,na.rm=TRUE),
        sum(data$edgeR.logFC<0 & data$edgeR.p.value<0.01,na.rm=TRUE),
        sum(data$edgeR.logFC<0 & data$edgeR.p.value<0.02,na.rm=TRUE),
        sum(data$edgeR.logFC<0 & data$edgeR.p.value<0.03,na.rm=TRUE),
        sum(data$edgeR.logFC<0 & data$edgeR.p.value<0.04,na.rm=TRUE),
        sum(data$edgeR.logFC<0 & data$edgeR.p.value<0.05,na.rm=TRUE),
        sum(data$edgeR.logFC<0 & data$edgeR.p.value<0.06,na.rm=TRUE),
        sum(data$edgeR.logFC<0 & data$edgeR.p.value<0.07,na.rm=TRUE),
        sum(data$edgeR.logFC<0 & data$edgeR.p.value<0.08,na.rm=TRUE),
        sum(data$edgeR.logFC<0 & data$edgeR.p.value<0.09,na.rm=TRUE),
        sum(data$edgeR.logFC<0 & data$edgeR.p.value<0.1,na.rm=TRUE),
        sum(data$edgeR.logFC<0 & data$edgeR.p.value<0.2,na.rm=TRUE),
        sum(data$edgeR.logFC<0 & data$edgeR.p.value<0.3,na.rm=TRUE),
        sum(data$edgeR.logFC<0 & data$edgeR.p.value<0.4,na.rm=TRUE),
        sum(data$edgeR.logFC<0 & data$edgeR.p.value<0.5,na.rm=TRUE),
        sum(data$edgeR.logFC<0 & data$edgeR.p.value<0.6,na.rm=TRUE),
        sum(data$edgeR.logFC<0 & data$edgeR.p.value<0.7,na.rm=TRUE),
        sum(data$edgeR.logFC<0 & data$edgeR.p.value<0.8,na.rm=TRUE),
        sum(data$edgeR.logFC<0 & data$edgeR.p.value<0.9,na.rm=TRUE),
        sum(data$edgeR.logFC<0 & data$edgeR.p.value<2,na.rm=TRUE))

    data.hyper.00001 <-
MEDIPS.mergeFrames(frames=subset(data,edgeR.logFC>0 &
edgeR.p.value<0.00001))
    data.hyper.00002 <-
MEDIPS.mergeFrames(frames=subset(data,edgeR.logFC>0 &
edgeR.p.value<0.00002))
    data.hyper.00003 <-
MEDIPS.mergeFrames(frames=subset(data,edgeR.logFC>0 &
edgeR.p.value<0.00003))
    data.hyper.00004 <-
MEDIPS.mergeFrames(frames=subset(data,edgeR.logFC>0 &
edgeR.p.value<0.00004))
```

```
    data.hyper.00005 <-
MEDIPS.mergeFrames(frames=subset(data,edgeR.logFC>0 &
edgeR.p.value<0.00005))
    data.hyper.00006 <-
MEDIPS.mergeFrames(frames=subset(data,edgeR.logFC>0 &
edgeR.p.value<0.00006))
    data.hyper.00007 <-
MEDIPS.mergeFrames(frames=subset(data,edgeR.logFC>0 &
edgeR.p.value<0.00007))
    data.hyper.00008 <-
MEDIPS.mergeFrames(frames=subset(data,edgeR.logFC>0 &
edgeR.p.value<0.00008))
    data.hyper.00009 <-
MEDIPS.mergeFrames(frames=subset(data,edgeR.logFC>0 &
edgeR.p.value<0.00009))
    data.hyper.0001 <-
MEDIPS.mergeFrames(frames=subset(data,edgeR.logFC>0 &
edgeR.p.value<0.0001))
    data.hyper.0002 <-
MEDIPS.mergeFrames(frames=subset(data,edgeR.logFC>0 &
edgeR.p.value<0.0002))
    data.hyper.0003 <-
MEDIPS.mergeFrames(frames=subset(data,edgeR.logFC>0 &
edgeR.p.value<0.0003))
    data.hyper.0004 <-
MEDIPS.mergeFrames(frames=subset(data,edgeR.logFC>0 &
edgeR.p.value<0.0004))
    data.hyper.0005 <-
MEDIPS.mergeFrames(frames=subset(data,edgeR.logFC>0 &
edgeR.p.value<0.0005))
    data.hyper.0006 <-
MEDIPS.mergeFrames(frames=subset(data,edgeR.logFC>0 &
edgeR.p.value<0.0006))
    data.hyper.0007 <-
MEDIPS.mergeFrames(frames=subset(data,edgeR.logFC>0 &
edgeR.p.value<0.0007))
    data.hyper.0008 <-
MEDIPS.mergeFrames(frames=subset(data,edgeR.logFC>0 &
edgeR.p.value<0.0008))
    data.hyper.0009 <-
MEDIPS.mergeFrames(frames=subset(data,edgeR.logFC>0 &
edgeR.p.value<0.0009))
    data.hyper.001 <-
MEDIPS.mergeFrames(frames=subset(data,edgeR.logFC>0 &
edgeR.p.value<0.001))
    data.hyper.002 <-
MEDIPS.mergeFrames(frames=subset(data,edgeR.logFC>0 &
edgeR.p.value<0.002))
    data.hyper.003 <-
MEDIPS.mergeFrames(frames=subset(data,edgeR.logFC>0 &
```

```
edgeR.p.value<0.003))
    data.hyper.004 <-
MEDIPS.mergeFrames(frames=subset(data,edgeR.logFC>0 &
edgeR.p.value<0.004))
    data.hyper.005 <-
MEDIPS.mergeFrames(frames=subset(data,edgeR.logFC>0 &
edgeR.p.value<0.005))
    data.hyper.006 <-
MEDIPS.mergeFrames(frames=subset(data,edgeR.logFC>0 &
edgeR.p.value<0.006))
    data.hyper.007 <-
MEDIPS.mergeFrames(frames=subset(data,edgeR.logFC>0 &
edgeR.p.value<0.007))
    data.hyper.008 <-
MEDIPS.mergeFrames(frames=subset(data,edgeR.logFC>0 &
edgeR.p.value<0.008))
    data.hyper.009 <-
MEDIPS.mergeFrames(frames=subset(data,edgeR.logFC>0 &
edgeR.p.value<0.009))
    data.hyper.01 <-
MEDIPS.mergeFrames(frames=subset(data,edgeR.logFC>0 &
edgeR.p.value<0.01))
    data.hyper.02 <-
MEDIPS.mergeFrames(frames=subset(data,edgeR.logFC>0 &
edgeR.p.value<0.02))
    data.hyper.03 <-
MEDIPS.mergeFrames(frames=subset(data,edgeR.logFC>0 &
edgeR.p.value<0.03))
    data.hyper.04 <-
MEDIPS.mergeFrames(frames=subset(data,edgeR.logFC>0 &
edgeR.p.value<0.04))
    data.hyper.05 <-
MEDIPS.mergeFrames(frames=subset(data,edgeR.logFC>0 &
edgeR.p.value<0.05))
    data.hyper.06 <-
MEDIPS.mergeFrames(frames=subset(data,edgeR.logFC>0 &
edgeR.p.value<0.06))
    data.hyper.07 <-
MEDIPS.mergeFrames(frames=subset(data,edgeR.logFC>0 &
edgeR.p.value<0.07))
    data.hyper.08 <-
MEDIPS.mergeFrames(frames=subset(data,edgeR.logFC>0 &
edgeR.p.value<0.08))
    data.hyper.09 <-
MEDIPS.mergeFrames(frames=subset(data,edgeR.logFC>0 &
edgeR.p.value<0.09))
    data.hyper.1 <-
MEDIPS.mergeFrames(frames=subset(data,edgeR.logFC>0 &
edgeR.p.value<0.1))
    data.hyper.2 <-
```

```
MEDIPS.mergeFrames(frames=subset(data,edgeR.logFC>0 &
edgeR.p.value<0.2))
    data.hyper.3 <-
MEDIPS.mergeFrames(frames=subset(data,edgeR.logFC>0 &
edgeR.p.value<0.3))
    data.hyper.4 <-
MEDIPS.mergeFrames(frames=subset(data,edgeR.logFC>0 &
edgeR.p.value<0.4))
    data.hyper.5 <-
MEDIPS.mergeFrames(frames=subset(data,edgeR.logFC>0 &
edgeR.p.value<0.5))
    data.hyper.6 <-
MEDIPS.mergeFrames(frames=subset(data,edgeR.logFC>0 &
edgeR.p.value<0.6))
    data.hyper.7 <-
MEDIPS.mergeFrames(frames=subset(data,edgeR.logFC>0 &
edgeR.p.value<0.7))
    data.hyper.8 <-
MEDIPS.mergeFrames(frames=subset(data,edgeR.logFC>0 &
edgeR.p.value<0.8))
    data.hyper.9 <-
MEDIPS.mergeFrames(frames=subset(data,edgeR.logFC>0 &
edgeR.p.value<0.9))
    data.hyper.all <-
MEDIPS.mergeFrames(frames=subset(data,edgeR.logFC>0 &
edgeR.p.value<2))

    data.hypo.00001 <-
MEDIPS.mergeFrames(frames=subset(data,edgeR.logFC<0 &
edgeR.p.value<0.00001))
    data.hypo.00002 <-
MEDIPS.mergeFrames(frames=subset(data,edgeR.logFC<0 &
edgeR.p.value<0.00002))
    data.hypo.00003 <-
MEDIPS.mergeFrames(frames=subset(data,edgeR.logFC<0 &
edgeR.p.value<0.00003))
    data.hypo.00004 <-
MEDIPS.mergeFrames(frames=subset(data,edgeR.logFC<0 &
edgeR.p.value<0.00004))
    data.hypo.00005 <-
MEDIPS.mergeFrames(frames=subset(data,edgeR.logFC<0 &
edgeR.p.value<0.00005))
    data.hypo.00006 <-
MEDIPS.mergeFrames(frames=subset(data,edgeR.logFC<0 &
edgeR.p.value<0.00006))
    data.hypo.00007 <-
MEDIPS.mergeFrames(frames=subset(data,edgeR.logFC<0 &
edgeR.p.value<0.00007))
    data.hypo.00008 <-
MEDIPS.mergeFrames(frames=subset(data,edgeR.logFC<0 &
```

```
edgeR.p.value<0.00008))
    data.hypo.00009 <-
MEDIPS.mergeFrames(frames=subset(data,edgeR.logFC<0 &
edgeR.p.value<0.00009))
    data.hypo.0001 <-
MEDIPS.mergeFrames(frames=subset(data,edgeR.logFC<0 &
edgeR.p.value<0.0001))
    data.hypo.0002 <-
MEDIPS.mergeFrames(frames=subset(data,edgeR.logFC<0 &
edgeR.p.value<0.0002))
    data.hypo.0003 <-
MEDIPS.mergeFrames(frames=subset(data,edgeR.logFC<0 &
edgeR.p.value<0.0003))
    data.hypo.0004 <-
MEDIPS.mergeFrames(frames=subset(data,edgeR.logFC<0 &
edgeR.p.value<0.0004))
    data.hypo.0005 <-
MEDIPS.mergeFrames(frames=subset(data,edgeR.logFC<0 &
edgeR.p.value<0.0005))
    data.hypo.0006 <-
MEDIPS.mergeFrames(frames=subset(data,edgeR.logFC<0 &
edgeR.p.value<0.0006))
    data.hypo.0007 <-
MEDIPS.mergeFrames(frames=subset(data,edgeR.logFC<0 &
edgeR.p.value<0.0007))
    data.hypo.0008 <-
MEDIPS.mergeFrames(frames=subset(data,edgeR.logFC<0 &
edgeR.p.value<0.0008))
    data.hypo.0009 <-
MEDIPS.mergeFrames(frames=subset(data,edgeR.logFC<0 &
edgeR.p.value<0.0009))
    data.hypo.001 <-
MEDIPS.mergeFrames(frames=subset(data,edgeR.logFC<0 &
edgeR.p.value<0.001))
    data.hypo.002 <-
MEDIPS.mergeFrames(frames=subset(data,edgeR.logFC<0 &
edgeR.p.value<0.002))
    data.hypo.003 <-
MEDIPS.mergeFrames(frames=subset(data,edgeR.logFC<0 &
edgeR.p.value<0.003))
    data.hypo.004 <-
MEDIPS.mergeFrames(frames=subset(data,edgeR.logFC<0 &
edgeR.p.value<0.004))
    data.hypo.005 <-
MEDIPS.mergeFrames(frames=subset(data,edgeR.logFC<0 &
edgeR.p.value<0.005))
    data.hypo.006 <-
MEDIPS.mergeFrames(frames=subset(data,edgeR.logFC<0 &
edgeR.p.value<0.006))
    data.hypo.007 <-
```

```
MEDIPS.mergeFrames(frames=subset(data,edgeR.logFC<0 &
edgeR.p.value<0.007))
    data.hypo.008 <-
MEDIPS.mergeFrames(frames=subset(data,edgeR.logFC<0 &
edgeR.p.value<0.008))
    data.hypo.009 <-
MEDIPS.mergeFrames(frames=subset(data,edgeR.logFC<0 &
edgeR.p.value<0.009))
    data.hypo.01 <-
MEDIPS.mergeFrames(frames=subset(data,edgeR.logFC<0 &
edgeR.p.value<0.01))
    data.hypo.02 <-
MEDIPS.mergeFrames(frames=subset(data,edgeR.logFC<0 &
edgeR.p.value<0.02))
    data.hypo.03 <-
MEDIPS.mergeFrames(frames=subset(data,edgeR.logFC<0 &
edgeR.p.value<0.03))
    data.hypo.04 <-
MEDIPS.mergeFrames(frames=subset(data,edgeR.logFC<0 &
edgeR.p.value<0.04))
    data.hypo.05 <-
MEDIPS.mergeFrames(frames=subset(data,edgeR.logFC<0 &
edgeR.p.value<0.05))
    data.hypo.06 <-
MEDIPS.mergeFrames(frames=subset(data,edgeR.logFC<0 &
edgeR.p.value<0.06))
    data.hypo.07 <-
MEDIPS.mergeFrames(frames=subset(data,edgeR.logFC<0 &
edgeR.p.value<0.07))
    data.hypo.08 <-
MEDIPS.mergeFrames(frames=subset(data,edgeR.logFC<0 &
edgeR.p.value<0.08))
    data.hypo.09 <-
MEDIPS.mergeFrames(frames=subset(data,edgeR.logFC<0 &
edgeR.p.value<0.09))
    data.hypo.1 <- MEDIPS.mergeFrames(frames=subset(data,edgeR.logFC<0
& edgeR.p.value<0.1))
    data.hypo.2 <- MEDIPS.mergeFrames(frames=subset(data,edgeR.logFC<0
& edgeR.p.value<0.2))
    data.hypo.3 <- MEDIPS.mergeFrames(frames=subset(data,edgeR.logFC<0
& edgeR.p.value<0.3))
    data.hypo.4 <- MEDIPS.mergeFrames(frames=subset(data,edgeR.logFC<0
& edgeR.p.value<0.4))
    data.hypo.5 <- MEDIPS.mergeFrames(frames=subset(data,edgeR.logFC<0
& edgeR.p.value<0.5))
    data.hypo.6 <- MEDIPS.mergeFrames(frames=subset(data,edgeR.logFC<0
& edgeR.p.value<0.6))
    data.hypo.7 <- MEDIPS.mergeFrames(frames=subset(data,edgeR.logFC<0
& edgeR.p.value<0.7))
    data.hypo.8 <- MEDIPS.mergeFrames(frames=subset(data,edgeR.logFC<0
```

```
& edgeR.p.value<0.8))
    data.hypo.9 <- MEDIPS.mergeFrames(frames=subset(data,edgeR.logFC<0
& edgeR.p.value<0.9))
    data.hypo.all <-
MEDIPS.mergeFrames(frames=subset(data,edgeR.logFC<0 &
edgeR.p.value<2))

    summary$dmr.fat.hyper.merged <- c(nrow(data.hyper.00001),
nrow(data.hyper.00002), nrow(data.hyper.00003), nrow(data.hyper.
00004), nrow(data.hyper.00005), nrow(data.hyper.00006),
nrow(data.hyper.00007), nrow(data.hyper.00008), nrow(data.hyper.
00009), nrow(data.hyper.0001), nrow(data.hyper.0002), nrow(data.hyper.
0003), nrow(data.hyper.0004), nrow(data.hyper.0005), nrow(data.hyper.
0006), nrow(data.hyper.0007), nrow(data.hyper.0008), nrow(data.hyper.
0009), nrow(data.hyper.001), nrow(data.hyper.002), nrow(data.hyper.
003), nrow(data.hyper.004), nrow(data.hyper.005), nrow(data.hyper.
006), nrow(data.hyper.007), nrow(data.hyper.008), nrow(data.hyper.
009), nrow(data.hyper.01), nrow(data.hyper.02), nrow(data.hyper.03),
nrow(data.hyper.04), nrow(data.hyper.05), nrow(data.hyper.06),
nrow(data.hyper.07), nrow(data.hyper.08), nrow(data.hyper.09),
nrow(data.hyper.1), nrow(data.hyper.2), nrow(data.hyper.3),
nrow(data.hyper.4), nrow(data.hyper.5), nrow(data.hyper.6),
nrow(data.hyper.7), nrow(data.hyper.8), nrow(data.hyper.9),
nrow(data.hyper.all))
    summary$dmr.fat.hypo.merged <- c(nrow(data.hypo.00001),
nrow(data.hypo.00002), nrow(data.hypo.00003), nrow(data.hypo.00004),
nrow(data.hypo.00005), nrow(data.hypo.00006), nrow(data.hypo.00007),
nrow(data.hypo.00008), nrow(data.hypo.00009), nrow(data.hypo.0001),
nrow(data.hypo.0002), nrow(data.hypo.0003), nrow(data.hypo.0004),
nrow(data.hypo.0005), nrow(data.hypo.0006), nrow(data.hypo.0007),
nrow(data.hypo.0008), nrow(data.hypo.0009), nrow(data.hypo.001),
nrow(data.hypo.002), nrow(data.hypo.003), nrow(data.hypo.004),
nrow(data.hypo.005), nrow(data.hypo.006), nrow(data.hypo.007),
nrow(data.hypo.008), nrow(data.hypo.009), nrow(data.hypo.01),
nrow(data.hypo.02), nrow(data.hypo.03), nrow(data.hypo.04),
nrow(data.hypo.05), nrow(data.hypo.06), nrow(data.hypo.07),
nrow(data.hypo.08), nrow(data.hypo.09), nrow(data.hypo.1),
nrow(data.hypo.2), nrow(data.hypo.3), nrow(data.hypo.4),
nrow(data.hypo.5), nrow(data.hypo.6), nrow(data.hypo.7),
nrow(data.hypo.8), nrow(data.hypo.9), nrow(data.hypo.all))

    significance.threshold.csv <-
write.table(summary,file="significance.threshold.csv")

  # Extracting significant DMRs (p<0.001)
    dmr.fat.tbt <-
subset(dmr.fat.TBT,edgeR.p.value<0.001,select=c(1:3,24,26))
    dmr.fat.tbt.csv <-
write.table(subset(dmr.fat.tbt,edgeR.p.value<0.001),file="dmr.fat.tbt.
csv")
```

```
# 2. Use of edgeR to identify significant TBT-dependent changes in F4
male adipose tissue transcriptome

  # Requirements: R (3.3), Bioconductor (3.3), Rsubread (1.22), and
edgeR (3.14)
  # Datasets (Gene Expression Omnibus: GSE105051):
RNA_DMSO_1.merged.sorted.bam (GSM2816958),
RNA_DMSO_2.merged.sorted.bam (GSM2816959),
RNA_DMSO_3.merged.sorted.bam (GSM2816960),
RNA_DMSO_4.merged.sorted.bam (GSM2816961), RNA_TBT_1.merged.sorted.bam
(GSM2816962), RNA_TBT_2.merged.sorted.bam (GSM2816963),
RNA_TBT_3.merged.sorted.bam (GSM2816964), RNA_TBT_4.merged.sorted.bam
(GSM2816965)

  # Mapping RNA-seq reads to Mmusculus mm10 gene annotation
    bam.files <- list.files(pattern=".merged.sorted.bam$")
    gtf.file <- ("mouse_refseq_anno.gtf")
    counts <- featureCounts(bam.files, annot.ext = gtf.file,
isGTFAnnotationFile = TRUE, GTF.featureType = "exon", GTF.attrType =
"gene_id", allowMultiOverlap = TRUE, isPairedEnd = FALSE, nthreads =
24, strandSpecific = 0)
    counts.df <- as.data.frame(counts$counts)
    colnames(counts.df) <- sub(".merged.sorted.bam", "",
colnames(counts.df))
    group <- factor(c(1,1,1,1,2,2,2,2))
    design <- model.matrix(~group)

  # Comparing RNA-seq read coverage for Mmusculus mm10 genes
    deg.fat.tbt.dge <- DGEList(counts = counts.df.deg.fat.tbt, group =
group)
    deg.fat.tbt.dge <- calcNormFactors(deg.fat.tbt.dge)
    deg.fat.tbt.dge <- estimateDisp(deg.fat.tbt.dge, design)
    deg.fat.tbt.fit <- glmQLFit(deg.fat.tbt.dge, design)
    deg.fat.tbt.qlf <- glmQLFTest(deg.fat.tbt.fit, coef = 2)
    topTags(deg.fat.tbt.qlf)
    write.table(cpm(deg.fat.tbt.dge), file =
"deg.fat.tbt_normalized_counts.txt", quote = FALSE, sep = "\t")
    write.table(topTags(deg.fat.tbt.qlf, n=1000000), file =
"deg.fat.tbt_DGEsummary.txt", quote = FALSE, sep = "\t")


# 3. Use of chromVar to study the similarity of F3 and F4 sperm
chromatin accessibility

  # Requirements: R (3.3), SICER, chromVar, and
BSgenome.Mmusculus.UCSC.mm10
  # Datasets (Gene Expression Omnibus: GSE105051):
ATAC_DMSO_1.merged.sorted.uniq.bam (GSM2816974),
ATAC_DMSO_2.merged.sorted.uniq.bam (GSM2816975),
```

```
ATAC_DMSO_3.merged.sorted.uniq.bam (GSM2816976),
ATAC_DMSO_4.merged.sorted.uniq.bam (GSM2816977),
ATAC_DMSO_5.merged.sorted.uniq.bam (GSM2816978),
ATAC_DMSO_6.merged.sorted.uniq.bam (GSM2816979),
ATAC_DMSO_7.merged.sorted.uniq.bam (GSM2816980),
ATAC_DMSO_8.merged.sorted.uniq.bam (GSM2816981),
ATAC_DMSO_9.merged.sorted.uniq.bam (GSM2816982),
ATAC_DMSO_10.merged.sorted.uniq.bam (GSM2816983),
ATAC_DMSO_11.merged.sorted.uniq.bam (GSM2816984),
ATAC_DMSO_12.merged.sorted.uniq.bam (GSM2816985),
ATAC_TBT_1.merged.sorted.uniq.bam (GSM2816986),
ATAC_TBT_2.merged.sorted.uniq.bam (GSM2816987),
ATAC_TBT_3.merged.sorted.uniq.bam (GSM2816988),
ATAC_TBT_4.merged.sorted.uniq.bam (GSM2816989),
ATAC_TBT_5.merged.sorted.uniq.bam (GSM2816990),
ATAC_TBT_6.merged.sorted.uniq.bam (GSM2816991),
ATAC_TBT_7.merged.sorted.uniq.bam (GSM2816992),
ATAC_TBT_8.merged.sorted.uniq.bam (GSM2816993),
ATAC_TBT_9.merged.sorted.uniq.bam (GSM2816994),
ATAC_TBT_10.merged.sorted.uniq.bam (GSM2816995),
ATAC_TBT_11.merged.sorted.uniq.bam (GSM2816996),
ATAC_TBT_12.merged.sorted.uniq.bam (GSM2816997)
  # peakfiles: SICER islands (see main text for further details),
Mmusculus mm10 isochores obtained from isoFinder

  # Mapping ATAC-seq reads with regard to SICER islands and Mmusculus
mm10 iscochores
    peaks <- getPeaks(peakfile)
    bamfiles <- c("ATAC_DMSO_1.merged.sorted.uniq.bam",
                  "ATAC_DMSO_2.merged.sorted.uniq.bam",
                  "ATAC_DMSO_3.merged.sorted.uniq.bam",
                  "ATAC_DMSO_4.merged.sorted.uniq.bam",
                  "ATAC_DMSO_5.merged.sorted.uniq.bam",
                  "ATAC_DMSO_6.merged.sorted.uniq.bam",
                  "ATAC_DMSO_7.merged.sorted.uniq.bam",
                  "ATAC_DMSO_8.merged.sorted.uniq.bam",
                  "ATAC_DMSO_9.merged.sorted.uniq.bam",
                  "ATAC_DMSO_10.merged.sorted.uniq.bam",
                  "ATAC_DMSO_11.merged.sorted.uniq.bam",
                  "ATAC_DMSO_12.merged.sorted.uniq.bam",
                  "ATAC_TBT_1.merged.sorted.uniq.bam",
                  "ATAC_TBT_2.merged.sorted.uniq.bam",
                  "ATAC_TBT_3.merged.sorted.uniq.bam",
                  "ATAC_TBT_4.merged.sorted.uniq.bam",
                  "ATAC_TBT_5.merged.sorted.uniq.bam",
                  "ATAC_TBT_6.merged.sorted.uniq.bam",
                  "ATAC_TBT_7.merged.sorted.uniq.bam",
                  "ATAC_TBT_8.merged.sorted.uniq.bam",
                  "ATAC_TBT_9.merged.sorted.uniq.bam",
                  "ATAC_TBT_10.merged.sorted.uniq.bam",
```

```
                    "ATAC_TBT_11.merged.sorted.uniq.bam",
                    "ATAC_TBT_12.merged.sorted.uniq.bam")

    cell.type <- c("DMSO", "DMSO", "DMSO", "DMSO", "DMSO", "DMSO",
"DMSO", "DMSO", "DMSO", "DMSO", "DMSO", "DMSO", "TBT", "TBT", "TBT",
"TBT", "TBT", "TBT", "TBT", "TBT", "TBT", "TBT", "TBT", "TBT")
    fragment_counts <- getCounts(bamfiles, peaks, paired =  FALSE,
by_rg = FALSE, format = "bam", colData = DataFrame(celltype =
cell.type))
    fragment_counts <- addGCBias(fragment_counts, genome =
BSgenome.Mmusculus.UCSC.mm10)
    counts_filtered <- filterSamples(fragment_counts, min_depth =
1500, min_in_peaks = 0.15, shiny = FALSE)
    counts_filtered <- filterPeaks(counts_filtered, non_overlapping =
TRUE)
    fragments_per_sample <- getFragmentsPerSample(counts_filtered)
    fragments_per_peak <- getFragmentsPerPeak(counts_filtered)
    dev <- computeDeviations(object = counts_filtered)

  # Calculating DMSO and TBT, F3 and F4 sample similarities for ATAC-
seq genomic distribution
    sample_cor <- getSampleCorrelation(dev, th=1.5)
    write.table(sample_cor, file = "sample_cor.txt", quote = FALSE,
sep = "\t")


# 4. Use of MEDIPS to identify significant TBT-dependent changes in F3
and F4 sperm chromatin accessibility

  # Requirements: R (3.3), Bioconductor (3.3), MEDIPS (1.22), and
BSgenome.Mmusculus.UCSC.mm10
  # Datasets (Gene Expression Omnibus: GSE105051):
ATAC_DMSO_1.merged.sorted.uniq.bam (GSM2816974),
ATAC_DMSO_2.merged.sorted.uniq.bam (GSM2816975),
ATAC_DMSO_3.merged.sorted.uniq.bam (GSM2816976),
ATAC_DMSO_4.merged.sorted.uniq.bam (GSM2816977),
ATAC_DMSO_5.merged.sorted.uniq.bam (GSM2816978),
ATAC_DMSO_6.merged.sorted.uniq.bam (GSM2816979),
ATAC_DMSO_7.merged.sorted.uniq.bam (GSM2816980),
ATAC_DMSO_8.merged.sorted.uniq.bam (GSM2816981),
ATAC_DMSO_9.merged.sorted.uniq.bam (GSM2816982),
ATAC_DMSO_10.merged.sorted.uniq.bam (GSM2816983),
ATAC_DMSO_11.merged.sorted.uniq.bam (GSM2816984),
ATAC_DMSO_12.merged.sorted.uniq.bam (GSM2816985),
ATAC_TBT_1.merged.sorted.uniq.bam (GSM2816986),
ATAC_TBT_2.merged.sorted.uniq.bam (GSM2816987),
ATAC_TBT_3.merged.sorted.uniq.bam (GSM2816988),
ATAC_TBT_4.merged.sorted.uniq.bam (GSM2816989),
ATAC_TBT_5.merged.sorted.uniq.bam (GSM2816990),
ATAC_TBT_6.merged.sorted.uniq.bam (GSM2816991),
```

```
ATAC_TBT_7.merged.sorted.uniq.bam (GSM2816992),
ATAC_TBT_8.merged.sorted.uniq.bam (GSM2816993),
ATAC_TBT_9.merged.sorted.uniq.bam (GSM2816994),
ATAC_TBT_10.merged.sorted.uniq.bam (GSM2816995),
ATAC_TBT_11.merged.sorted.uniq.bam (GSM2816996),
ATAC_TBT_12.merged.sorted.uniq.bam (GSM2816997)
  # peakfiles: SICER islands (see main text for further details),
Mmusculus mm10 isochores obtained from isoFinder

  # Setting MEDIPS parameters
    BSgenome = "BSgenome.Mmusculus.UCSC.mm10"
    uniq = 1e-3
    extend = 300
    shift = 0
    ws = 100
    selectedchromosomes = c("chr1", "chr2", "chr3", "chr4", "chr5",
"chr6", "chr7", "chr8", "chr9", "chr10", "chr11", "chr12", "chr13",
"chr14", "chr15", "chr16", "chr17", "chr18", "chr19", "chrX", "chrY")
    rois <- read.table("peakfiles")
    colnames(rois) <- c("chr", "start", "end", "ID")

  # Creating MEDIPS sets from bam files
    f3.DMSO1.ATAC <- MEDIPS.createROIset(file =
"ATAC_DMSO_1.merged.sorted.uniq.bam", ROI = rois, BSgenome = BSgenome,
extend = extend, shift = shift, uniq = uniq, chr.select =
selectedchromosomes)
    f3.DMSO2.ATAC <- MEDIPS.createROIset(file =
"ATAC_DMSO_2.merged.sorted.uniq.bam", ROI = rois, BSgenome = BSgenome,
extend = extend, shift = shift, uniq = uniq, chr.select =
selectedchromosomes)
    f3.DMSO3.ATAC <- MEDIPS.createROIset(file =
"ATAC_DMSO_3.merged.sorted.uniq.bam", ROI = rois, BSgenome = BSgenome,
extend = extend, shift = shift, uniq = uniq, chr.select =
selectedchromosomes)
    f3.DMSO4.ATAC <- MEDIPS.createROIset(file =
"ATAC_DMSO_4.merged.sorted.uniq.bam", ROI = rois, BSgenome = BSgenome,
extend = extend, shift = shift, uniq = uniq, chr.select =
selectedchromosomes)
    f3.DMSO5.ATAC <- MEDIPS.createROIset(file =
"ATAC_DMSO_5.merged.sorted.uniq.bam", ROI = rois, BSgenome = BSgenome,
extend = extend, shift = shift, uniq = uniq, chr.select =
selectedchromosomes)
    f3.DMSO6.ATAC <- MEDIPS.createROIset(file =
"ATAC_DMSO_6.merged.sorted.uniq.bam", ROI = rois, BSgenome = BSgenome,
extend = extend, shift = shift, uniq = uniq, chr.select =
selectedchromosomes)
    f3.DMSO.ATAC <- c(f3.DMSO1.ATAC, f3.DMSO2.ATAC, f3.DMSO3.ATAC,
f3.DMSO4.ATAC, f3.DMSO5.ATAC, f3.DMSO6.ATAC)

    f3.TBT1.ATAC <- MEDIPS.createROIset(file =
```

```
"ATAC_TBT_1.merged.sorted.uniq.bam", ROI = rois, BSgenome = BSgenome,
extend = extend, shift = shift, uniq = uniq, chr.select =
selectedchromosomes)
    f3.TBT2.ATAC <- MEDIPS.createROIset(file =
"ATAC_TBT_2.merged.sorted.uniq.bam", ROI = rois, BSgenome = BSgenome,
extend = extend, shift = shift, uniq = uniq, chr.select =
selectedchromosomes)
    f3.TBT3.ATAC <- MEDIPS.createROIset(file =
"ATAC_TBT_3.merged.sorted.uniq.bam", ROI = rois, BSgenome = BSgenome,
extend = extend, shift = shift, uniq = uniq, chr.select =
selectedchromosomes)
    f3.TBT4.ATAC <- MEDIPS.createROIset(file =
"ATAC_TBT_4.merged.sorted.uniq.bam", ROI = rois, BSgenome = BSgenome,
extend = extend, shift = shift, uniq = uniq, chr.select =
selectedchromosomes)
    f3.TBT5.ATAC <- MEDIPS.createROIset(file =
"ATAC_TB_5.merged.sorted.uniq.bam", ROI = rois, BSgenome = BSgenome,
extend = extend, shift = shift, uniq = uniq, chr.select =
selectedchromosomes)
    f3.TBT6.ATAC <- MEDIPS.createROIset(file =
"ATAC_TBT_6.merged.sorted.uniq.bam", ROI = rois, BSgenome = BSgenome,
extend = extend, shift = shift, uniq = uniq, chr.select =
selectedchromosomes)
    f3.TBT.ATAC <- c(f3.TBT1.ATAC, f3.TBT2.ATAC, f3.TBT3.ATAC,
f3.TBT4.ATAC, f3.TBT5.ATAC, f3.TBT6.ATAC)

    f4.DMSO1.ATAC <- MEDIPS.createROIset(file =
"ATAC_DMSO_7.merged.sorted.uniq.bam", ROI = rois, BSgenome = BSgenome,
extend = extend, shift = shift, uniq = uniq, chr.select =
selectedchromosomes)
    f4.DMSO2.ATAC <- MEDIPS.createROIset(file =
"ATAC_DMSO_8.merged.sorted.uniq.bam", ROI = rois, BSgenome = BSgenome,
extend = extend, shift = shift, uniq = uniq, chr.select =
selectedchromosomes)
    f4.DMSO3.ATAC <- MEDIPS.createROIset(file =
"ATAC_DMSO_9.merged.sorted.uniq.bam", ROI = rois, BSgenome = BSgenome,
extend = extend, shift = shift, uniq = uniq, chr.select =
selectedchromosomes)
    f4.DMSO4.ATAC <- MEDIPS.createROIset(file =
"ATAC_DMSO_10.merged.sorted.uniq.bam", ROI = rois, BSgenome =
BSgenome, extend = extend, shift = shift, uniq = uniq, chr.select =
selectedchromosomes)
    f4.DMSO5.ATAC <- MEDIPS.createROIset(file =
"ATAC_DMSO_11.merged.sorted.uniq.bam", ROI = rois, BSgenome =
BSgenome, extend = extend, shift = shift, uniq = uniq, chr.select =
selectedchromosomes)
    f4.DMSO6.ATAC <- MEDIPS.createROIset(file =
"ATAC_DMSO_12.merged.sorted.uniq.bamm", ROI = rois, BSgenome =
BSgenome, extend = extend, shift = shift, uniq = uniq, chr.select =
selectedchromosomes)
```

```
    f4.DMSO.ATAC <- c(f4.DMSO1.ATAC, f4.DMSO2.ATAC, f4.DMSO3.ATAC,
f4.DMSO4.ATAC, f4.DMSO5.ATAC, f4.DMSO6.ATAC)

    f4.TBT1.ATAC <- MEDIPS.createROIset(file =
"ATAC_TBT_7.merged.sorted.uniq.bam", ROI = rois, BSgenome = BSgenome,
extend = extend, shift = shift, uniq = uniq, chr.select =
selectedchromosomes)
    f4.TBT2.ATAC <- MEDIPS.createROIset(file =
"ATAC_TBT_8.merged.sorted.uniq.bam", ROI = rois, BSgenome = BSgenome,
extend = extend, shift = shift, uniq = uniq, chr.select =
selectedchromosomes)
    f4.TBT3.ATAC <- MEDIPS.createROIset(file =
"ATAC_TBT_9.merged.sorted.uniq.bam", ROI = rois, BSgenome = BSgenome,
extend = extend, shift = shift, uniq = uniq, chr.select =
selectedchromosomes)
    f4.TBT4.ATAC <- MEDIPS.createROIset(file =
"ATAC_TBT_10.merged.sorted.uniq.bam", ROI = rois, BSgenome = BSgenome,
extend = extend, shift = shift, uniq = uniq, chr.select =
selectedchromosomes)
    f4.TBT5.ATAC <- MEDIPS.createROIset(file =
"ATAC_TBT_11.merged.sorted.uniq.bam", ROI = rois, BSgenome = BSgenome,
extend = extend, shift = shift, uniq = uniq, chr.select =
selectedchromosomes)
    f4.TBT6.ATAC <- MEDIPS.createROIset(file =
"ATAC_TBT_12.merged.sorted.uniq.bam", ROI = rois, BSgenome = BSgenome,
extend = extend, shift = shift, uniq = uniq, chr.select =
selectedchromosomes)
    f4.TBT.ATAC <- c(f4.TBT1.ATAC, f4.TBT2.ATAC, f4.TBT3.ATAC,
f4.TBT4.ATAC, f4.TBT5.ATAC, f4.TBT6.ATAC)

  # Comparing ATAC-seq read coverage for SICER islands and Mmusculus
mm10 isochores
    f3.datac <- MEDIPS.meth(MSet1 = f3.TBT.ATAC, MSet2 = f3.DMSO.ATAC,
CSet = NULL, p.adj = "fdr", diff.method = "edgeR", MeDIP = FALSE, CNV
= FALSE, minRowSum = 10, diffnorm = "quantile")
    f3.datac.csv <- write.table(f3.datac,file="f3.datac.csv")

    f4.datac <- MEDIPS.meth(MSet1 = f4.TBT.ATAC, MSet2 = f4.DMSO.ATAC,
CSet = NULL, p.adj = "fdr", diff.method = "edgeR", MeDIP = FALSE, CNV
= FALSE, minRowSum = 10, diffnorm = "quantile")
    f4.datac.csv <- write.table(f4.datac,file="f4.datac.csv")
```
