# Supplementary material for: Ancestral perinatal obesogen exposure results in a transgenerational thrifty phenotype in mice
Source: Nat Commun. 2017 Dec 8;8:2012. doi: 10.1038/s41467-017-01944-z (PMC5722856; doi:10.1038/s41467-017-01944-z)
Supplement: Supplementary file 3 — Description of Additional Supplementary Files [file 41467_2017_1944_MOESM3_ESM.pdf]

Description of Additional Supplementary Files

File Name: Supplementary Data 1
Description: Summary of TBT-dependent DNA methylome and transcriptome variation in F4 male gonadal adipose tissue using different thresholds of statistical significance.

File Name: Supplementary Data 2
Description: TBT-dependent DNA methylome variation for F4 male gonadal adipose tissue.

File Name: Supplementary Data 3
Description: Genes associated to genomic structures defined using TBT-dependent DNA methylome variation for F4 male gonadal adipose tissue.

File Name: Supplementary Data 4
Description: iso-Differentially Methylated Blocks (isoDMBs) defined using TBT-dependent DNA methylome variation for F4 male gonadal adipose tissue.

File Name: Supplementary Data 5
Description: Results from Monte Carlo-Wilcoxon matched-pairs signed-ranks tests to study the coordination between TBT-dependent DNA methylome and transcriptome variation in F4 male gonadal adipose tissue.

File Name: Supplementary Data 6
Description: Results from Monte Carlo tests for the enrichment of groups of at least two TBTdependent differentially expressed genes (DEGs) within iso-Differentially Methylated Blocks (isoDMBs) for F4 male gonadal adipose tissue.

File Name: Supplementary Data 7
Description: Results from Monte Carlo simulations for the overlap of F4 male gonadal adipose tissue isoDMBs and mouse genes and isochores.

File Name: Supplementary Data 8
Description: Functional gene ontology (GO) analysis for genes spanning mouse isochores.

File Name: Supplementary Data 9
Description: isoDMB genes showing significant signs of coordination for TBT-dependent DNA methylome and transcriptome variation in F4 male gonadal adipose tissue.

File Name: Supplementary Data 10
Description: Functional gene ontology (GO) analysis for isoDMB genes showing significant signs of coordination for TBT-dependent DNA methylome and transcriptome variation in F4 male gonadal adipose tissue.

File Name: Supplementary Data 11
Description: . Genomic regions showing significant TBT-dependent chromatin accessibility variation in F3 and F4 sperm.

File Name: Supplementary Data 12
Description: Sample efficiencies for analyses of TBT-dependent chromatin accessibility variation in F3 and F4 sperm using ATAC-seq.

File Name: Supplementary Data 13
Description: Pearson correlations for F3 and F4, DMSO and TBT sperm samples with regard to their genomic distribution of ATAC-seq reads.

File Name: Supplementary Data 14
Description: Chi-square tests for the distribution of DMSO and TBT, or F3 and F4 samples with regard to main clusters and subclusters of chromVar base composition- and SICER-guided ATAC-seq dendrograms.

File Name: Supplementary Data 15
Description: Results of Monte Carlo tests for the distribution of genomic regions showing significant TBT-dependent chromatin accessibility variation in F3 and F4 sperm with regard to their base composition.

File Name: Supplementary Data 16
Description: Results from Monte Carlo simulations for the overlap of genomic regions showing significant TBT-dependent chromatic accessibility variation in F3 and F4 sperm and significant TBT-dependent DNA methylome variation in F4 male gonadal adipose tissue.

File Name: Supplementary Data 17
Description: Results from Monte Carlo test for the fraction of genomic regions with significant TBTdependent chromatin accessibility variation in F3 and F4 sperm and the same direction of change.

File Name: Supplementary Data 18
Description: Genes spanned by genomic regions showing significant TBT-dependent chromatin accessibility variation in F3 or F4 sperm.

File Name: Supplementary Data 19
Description: Functional gene ontology (GO) analysis for genes spanned by genomic regions with significant TBT-dependent chromatin accessibility variation in F3 and F4 sperm.

File Name: Supplementary Data 20
Description: Results from Monte Carlo simulations for the overlap of genomic regions showing significant TBT-dependent chromatin accessibility variation in F3 and F4 sperm and the same direction of change.

File Name: Supplementary Data 21
Description: Functional gene ontology (GO) analysis for genes spanned by the overlap between genomic regions with significant TBT-dependent chromatin accessibility variation in F3 and F4 sperm and the same direction of change.
